# BERT-Enhanced HyperGAT with Siamese Networks and Reference Answer Set for Automated Short-Answer Scoring

**DOI:** 10.3390/bs16060946

**Published:** 2026-06-09

**Authors:** Chen Liu, Xiaofen Wan, Zhihao Ni, Sheng Su, Chunhua Kang

**Affiliations:** Zhejiang Philosophy and Social Science Laboratory for the Mental Health and Crisis Intervention of Children and Adolescents, Zhejiang Normal University, Jinhua 321004, China; chenliu@zjnu.edu.cn (C.L.); 15751868735@163.com (X.W.); 17321424869@163.com (Z.N.); sus473830@gmail.com (S.S.)

**Keywords:** automated short-answer grading, hypergraph attention network, BERT, Siamese neural network, reference answer set

## Abstract

This paper proposes a novel framework, HyperGAT-BERT-RAS, that integrates (1) a HyperGraph Attention Network (HyperGAT) with BERT for enhanced semantic representation; (2) a Reference Answer Set (RAS) constructed via clustering of full-score answers; and (3) Siamese Neural Networks (SNNs) for similarity-based scoring. Experiments on the Ohsumed and ASAP-5 datasets demonstrate that (i) HyperGAT-BERT achieves 72.95% accuracy on Ohsumed text classification, outperforming baseline HyperGAT by 3.28%, and (ii) the full HyperGAT-BERT-RAS achieves 78.66% accuracy and 0.7806 F1-score, with RAS contributing the most to performance gains. These improvements suggest the potential for more reliable scoring of diverse student answers, reduced teacher grading burden, and enhanced feasibility of AI-assisted formative assessment in real classrooms, although empirical validation with teachers and students is needed.

## 1. Introduction

In everyday classroom practice, teachers spend countless hours grading short-answer questions, a task that is not only time-consuming but also prone to inconsistency due to fatigue or subjective judgment. This burden limits teachers’ ability to provide timely, personalized feedback, which is critical for student learning. While automated short-answer grading (ASAG) offers a promising solution, existing methods struggle with two practical challenges that hinder their adoption in real classrooms. First, the diversity of student answers. The same correct idea can be expressed in countless ways, yet most automated scoring models rely on limited reference answers, leading to frequent misclassifications (Tan et al., 2022). For example, in a biology short-answer question, some students may express “mRNA leaves the nucleus” as “mRNA goes out of the nucleus”—semantically correct but easily flagged as incorrect by keyword-matching systems. Second, the ambiguity of score boundaries. Teachers often encounter partially correct or creatively worded answers that fall into the “gray area” between score levels, making precise classification difficult. These challenges not only reduce scoring accuracy but also undermine teachers’ trust in automated grading systems.

To address these challenges, researchers have explored various approaches to improving ASAG. The following sections review prior work on text classification methods, reference answer set construction, leading to the formulation of our research hypotheses.

### 1.1. Text Classification Methods and Their Educational Limitations

In automated scoring research, text classification serves as the core technical framework. Early studies employed machine learning methods that relied on manually engineered features to build scoring models (Kumar et al., 2019; Saha et al., 2018; Sultan et al., 2016). These approaches are not only labor-intensive but also struggle to capture complex semantic relationships in student answers. Deep learning techniques, such as convolutional neural networks (CNNs) and long short-term memory networks (LSTMs), reduce the need for manual feature extraction but often fail to account for global semantic interactions (Alikaniotis et al., 2016; Huang et al., 2018; Riordan et al., 2017).

Recent studies have introduced graph convolutional networks (GCNs) to model global semantic structures in student answers (Tan et al., 2023). However, GCNs assume uniform importance among adjacent nodes, neglecting variations in how different words contribute to meaning (Gilmer et al., 2017). More importantly, traditional graphs are limited to pairwise connections, making it difficult to capture complex multi-word interactions in student answers. Hypergraphs address this limitation by allowing a single edge (hyperedge) to connect any number of nodes, enabling more effective modeling of high-order semantic relationships (Feng et al., 2019; Kim et al., 2020). Ding et al. (2020) proposed the HyperGraph Attention Network (HyperGAT), which achieved superior performance on text classification tasks compared to traditional methods, though its accuracy on the Ohsumed medical dataset remained modest at 0.69, indicating room for improvement.

Meanwhile, the emergence of pre-trained language models such as BERT has brought significant advances in text understanding. Unlike static word embeddings, BERT generates dynamic, context-dependent representations, effectively resolving polysemy (Devlin et al., 2019). However, BERT alone struggles to capture the structured semantic relationships between student answers and reference answers. The integration of large language models with graph-based knowledge representations has recently gained attention in educational technology research. For instance, Dobriţa and Oprea (2025) proposed an NLP-driven e-learning platform that combines LLMs with graph databases (Neo4j) to provide personalized career guidance based on user-uploaded documents, extracting skills and technologies using BERT, and generating embeddings with FAISS for similarity matching. This work further motivates our approach of combining BERT’s semantic understanding with HyperGAT’s relational modeling for educational assessment tasks, as both share the core idea of integrating language models with structured knowledge representations to enhance personalized learning experiences.

Therefore, Study 1 proposes integrating BERT with HyperGAT to develop a HyperGAT-BERT framework, with the goal of improving text classification accuracy. This design rationale is based on the premise that combining BERT’s contextual awareness with HyperGAT’s ability to model high-order relationships will better represent student answer semantics and thereby enhance automated scoring performance. The empirical evaluation below validates this design choice.

### 1.2. Reference Answer Sets

The coverage of reference answers is a critical factor affecting ASAG accuracy (Burrows et al., 2015; Valenti et al., 2003). In practice, the diversity of student answers often exceeds the coverage of predefined reference sets, leading to many correct answers being incorrectly flagged as wrong. This problem is particularly acute for open-ended questions (Tan et al., 2022).

**Figure 1:**
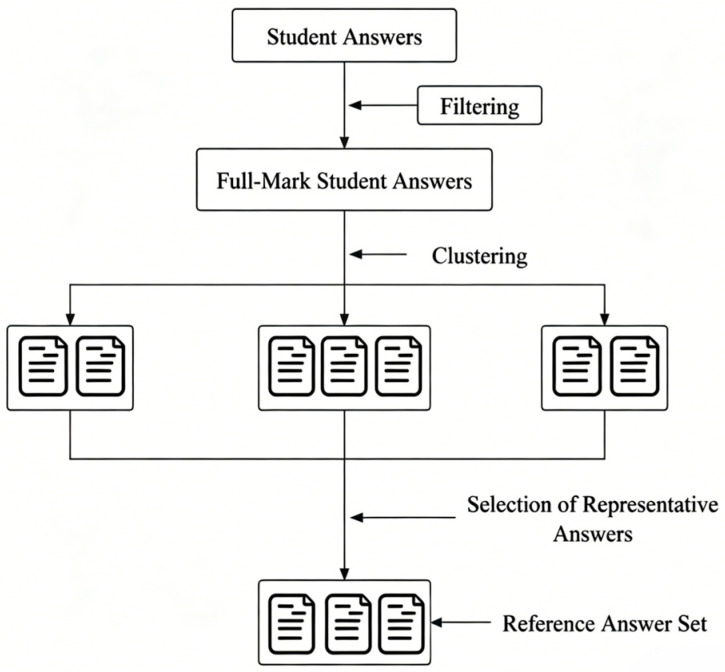
Construction of reference answer set via clustering of student answers.

To address this issue, researchers have explored ways to construct more comprehensive reference answer sets. Lan et al. (2015) proposed clustering student answers and having experts select representative examples from each cluster, streamlining the scoring process. Marvaniya et al. (2018) further clustered answers by score level, selecting representative answers from each level. Specifically, reference answer set construction involves two key steps (Tan et al., 2019): (1) clustering analysis to identify distinct answer patterns, with each cluster representing a unique response type; and (2) selecting one or more prototypical answers from each cluster to compile the reference answer set, as illustrated in Figure 1.

**Figure 2:**
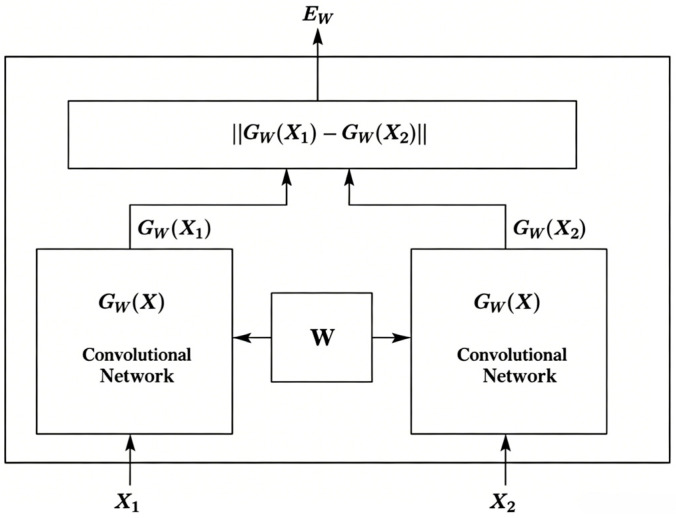
Siamese neural network architecture for answer similarity computation.

Furthermore, Siamese Neural Networks (SNNs), initially proposed for tasks such as face recognition, signature verification, and similarity learning (Bromley et al., 1993), have recently been adapted to educational applications, particularly in automated scoring of constructed-response items. The architecture comprises twin subnetworks that share identical parameters (e.g., weights and biases) but process two distinct input samples. As shown in Figure 2, this design enables SNNs to effectively quantify similarity or dissimilarity between paired inputs through comparative analysis.

Based on this analysis, Study 2 proposes constructing a Reference Answer Set (RAS) via clustering and integrating it with SNNs to form the HyperGAT-BERT-RAS framework. This design rationale assumes that the RAS will cover more diverse answer patterns, while the SNN will more precisely compute semantic similarity between student answers and reference answers. Ablation experiments are conducted to empirically validate the contribution of RAS to scoring accuracy and to assess this design choice.

### 1.3. Novel Contributions of This Work

Based on the above analysis, this study makes the following three contributions: (1) Technical integration. We propose a joint architecture combining BERT’s contextualized representations with HyperGAT’s hypergraph structure. On the Ohsumed text classification benchmark, this integration significantly outperforms BERT and HyperGAT baselines, demonstrating its effectiveness for semantic representation. For ASAG, the core technical contribution is the RAS-guided Siamese scoring mechanism. (2) RAS-guided Siamese scoring. Unlike prior work that uses RAS only for answer selection, we embed RAS directly into a Siamese architecture to compute fine-grained similarity scores. (3) Potential educational significance. By improving scoring accuracy and consistency, this study suggests the potential to reduce teacher grading burden, support formative assessment implementation, and provide a reliable tool for AI-assisted assessment in real classrooms.

## 2. HyperGAT-BERT Framework and Performance Validation

### 2.1. HyperGAT-BERT Architecture

**Figure 3:**
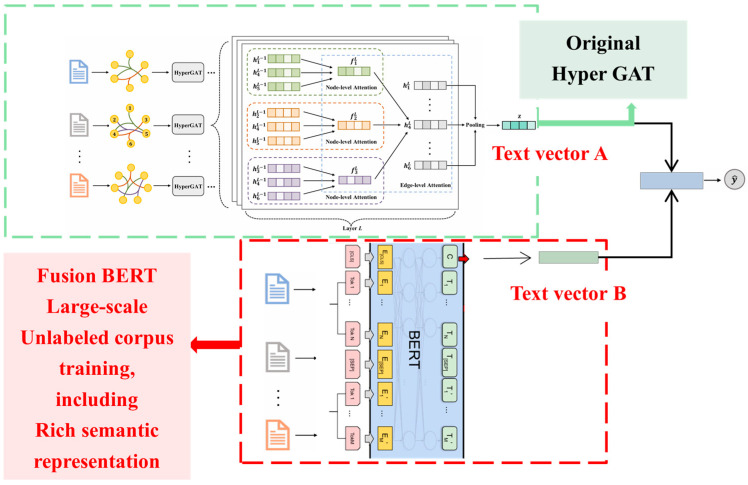
HyperGAT-BERT framework.

The HyperGAT framework incorporates a text hypergraph encoding syntactic and semantic information, along with dual attention mechanisms at both node and hyperedge levels (Ding et al., 2020). In this study, the proposed HyperGAT-BERT similarly treats each word as a hypernode and models contextual syntactic and semantic relationships through hyperedges. Multi-relational hyperedges, specifically syntactic hyperedges (encoding word order) and semantic hyperedges (encoding meaning), are defined following the original methodology. After constructing these hyperedges, dual attention mechanisms are employed to capture high-order word interactions while emphasizing critical information at varying granularities during node representation learning. For each document, node representations across the hypergraph are computed post-attention, followed by a mean pooling operation to derive the text representation. The BERT model, pretrained on large-scale unlabeled corpora, generates text embeddings rich in semantic information, which can be directly transferred to downstream tasks (Devlin et al., 2019). In this framework, text inputs are processed through BERT to obtain sequence representations, which are concatenated with HyperGAT-derived text representations (denoted as Concat). This fused document representation (Z) is then fed into a softmax layer for classification. The architecture is illustrated in Figure 3, where the green dashed box represents the original HyperGAT method (Ding et al., 2020), and the combined red-green components depict the BERT-enhanced HyperGAT-BERT framework.

The following is a mathematical formalization of the above architecture: 

Let $x=[w_{1},w_{2},\ldots ,w_{n}]$ denote an input document with n words. The hypergraph branch first constructs a text hypergraph where nodes correspond to words and hyperedges encode syntactic and semantic relationships. Through dual attention mechanisms at node and hyperedge levels, this branch produces a document representation $h_{\mathrm{Hyper}}\in R^{d_{h}}$, where $d_{h}$ is the embedding dimension. 

Simultaneously, the BERT branch encodes the same input using a pretrained BERT model, producing a contextualized representation $h_{\mathrm{BERT}}\in R^{d_{b}}$, where $d_{b}$ is the hidden dimension of BERT (e.g., 768 for BERT-base).

The two representations are concatenated to form the fused document representation:

(1) $$Z=[h_{\mathrm{Hyper}};h_{\mathrm{BERT}}]\in R^{d_{h}+d_{b}}$$

Finally, Z is passed through a softmax layer for classification:

(2) $$\hat{y}=\mathrm{softmax}(W_{c}Z+b_{c})$$

where $W_{c}\in R^{C\times (d_{h}+d_{b})}$ is the weight matrix, $b_{c}\in R^{C}$ is the bias term, and C is the num-ber of classes in the classification task.

### 2.2. Text Classification Performance Validation

#### 2.2.1. Ohsumed Dataset

The Ohsumed corpus comprises medical literature abstracts sourced from the MEDLINE database, a critical repository curated by the U.S. National Library of Medicine (http://disi.unitn.it/moschitti/corpora.htm, accessed on 6 May 2023). This study utilizes 13,929 cardiovascular disease abstracts selected from the first 20,000 articles published in 1991. Each document in the dataset is associated with one or more category labels spanning 23 disease classes. To ensure label consistency, documents with multiple category assignments were excluded, resulting in a final subset of 7400 single-label documents. In each experimental run, 80% of the training samples are randomly selected for model training, while the remaining 20% are reserved for validation. Table 1 summarizes the basic characteristics of the Ohsumed dataset. It is important to clarify that the Ohsumed dataset is used here only to evaluate the basic text classification performance of the HyperGAT-BERT model, not as an ASAG task. This serves as a necessary validation step to assess whether the integration of BERT with HyperGAT improves semantic representation capability before applying the model to the actual short-answer grading task on ASAP-5. Therefore, the results on Ohsumed should be interpreted as evidence of the model’s general text classification ability rather than its ASAG-specific performance.

#### 2.2.2. Experimental Design

To investigate whether HyperGAT (Ding et al., 2020) and the proposed HyperGAT-BERT can better represent texts and improve text classification performance, this study conducted comprehensive experiments to evaluate model performance on text classification tasks. The HyperGAT-BERT model was compared against five categories of baseline models, including word embedding-based methods (SWEM), sequence-based methods (CNN-non-static), a pre-trained model based on bidirectional Transformer (BERT), and graph-based methods (Text-level GNN and HyperGAT). Data analysis is conducted using Python3.

Hyperparameter Tuning. The optimal hyperparameters were determined via grid search over the following ranges: embedding dimensions {128, 200, 300, 400}; learning rates { 1 × 10^−4^, 5 × 10^−4^, 1 × 10^−3^}; dropout rates {0.2, 0.3, 0.4}; batch sizes {8, 16, 32}. The validation set (10% of the training data) was used for model selection. The best configuration was identified as embedding dimension = 300, learning rate = 0.001, dropout = 0.3, and batch size = 16. Sensitivity analysis for the embedding dimension is presented in Figure 6. For all models, we employed early stopping with a patience of 5 epochs, meaning that training would terminate if validation performance did not improve for five consecutive epochs. By default, the maximum number of epochs is set to 100. The Adam optimizer was used with weight decay set to 1 × 10^−6^, and L2 regularization was applied with a coefficient of 1 × 10^−6^.

Reproducibility and Statistical Validation. The pre-trained BERT model used in all experiments is bert-base-uncased (12-layer, 110M parameters) from the HuggingFace Transformers library. The BertTokenizer was used with a maximum sequence length of 512 tokens; longer sequences were truncated, and shorter sequences were padded. During training, the BERT branch was fine-tuned jointly with the rest of the network (i.e., all BERT parameters were updated via backpropagation, not frozen). To ensure robust statistical evaluation, each model was trained and evaluated 10 times with different random seeds. The initial random seed was set to 666, and subsequent runs used seeds incremented by one (i.e., 666, 667, 668, …, 675). This approach allows us to report mean accuracy and standard deviation across multiple runs and conduct statistical significance testing (paired-sample *t*-tests) between models.

Training Time. The training time varied across models depending on the model complexity. For the baseline models (BERT, SWEM, CNN-non-static, Text-level GNN, HyperGAT), training time ranged from approximately 1 to 3 min per run. For the proposed HyperGAT-BERT model, training time was approximately 6 min per run. All experiments were conducted on a single NVIDIA Tesla V100 (32GB) GPU.

The evaluation metrics are Recall (R), Precision (P), Accuracy (ACC), and F1-score (F1). Recall (R) is defined as the proportion of actual positive samples correctly identified. Precision (P) evaluates the proportion of correctly predicted positive samples among those identified as positive. Accuracy (ACC) represents the proportion of correctly classified samples among all samples. The F1-score balances precision and recall. Although recall (R) is a conventional metric for classification tasks, we do not report it separately in our results. The F1-score, as the harmonic mean of precision and recall, already captures the balance between the two. Reporting recall alone would provide redundant information without additional insight into model performance for ASAG. Therefore, only P, ACC, and F1 are presented in the following result tables. The formulas of R, P, ACC, and F1 are as follows (3)–(6):

(3) $$R\;=\;\frac{\mathrm{TP}}{\;\mathrm{TP}\;+\;FN}$$

(4) $$P\;=\;\frac{\mathrm{TP}}{\mathrm{TP}\;+\;\mathrm{FP}}$$

(5) $$F1\;=\;\frac{2\;\times \;P\;\times \;R}{P\;+\;R}$$

(6) $$\mathrm{ACC}\;=\;\frac{\mathrm{TP}\;+\;\mathrm{TN}}{\mathrm{TP}\;+\;\mathrm{TN}\;+\;\mathrm{FP}\;+\;\mathrm{FN}}$$

where TP (True Positive), TN (True Negative), FP (False Positive), and FN (False Negative) are defined as follows: TP, the number of samples correctly predicted as the positive class; TN, the number of samples correctly predicted as the negative class; FP, the number of samples incorrectly predicted as the positive class; FN, the number of samples incorrectly predicted as the negative class.

#### 2.2.3. Experimental Results

To facilitate comparison with the results reported in Ding et al. (2020), we also report only the mean accuracy (ACC) and standard deviation (SD) for each model in this section. The results are presented in Table 2. The source code for the baseline methods (SWEM, CNN-non-static, etc.) is available at the repository provided by Ding et al. (2020): https://github.com/kaize0409/HyperGAT, accessed on 6 May 2023. The source code for our proposed HyperGAT-BERT and HyperGAT-BERT-RAS is available at our anonymized repository: https://anonymous.4open.science/r/HyperGAT-BERT-RAS/, accessed after 21 May 2026.

As shown in Table 2, graph-based methods (Text-level GNN and HyperGAT) achieved superior performance compared to word embedding-based and sequence-based methods. This result indicates that graph structures can better capture long-distance word interactions, thereby improving text classification performance. Compared to the baseline models, HyperGAT-BERT achieved the highest accuracy (0.7295) among all compared models, surpassing SWEM by 9.8%, CNN-non-static by 14.5%, Text-level GNN by 3.5%, HyperGAT by 3.28%, and BERT by 2.93%. This improvement demonstrates that integrating BERT’s rich semantic representations enhances HyperGAT’s text representation capability, which is significant for text classification tasks. To further validate the effectiveness of HyperGAT-BERT, we compared it with results reported in existing literature on the same dataset. Lv et al. (2024) reported that their RB-GAT model achieved 0.7148 accuracy on the Ohsumed dataset. However, as their experimental settings (e.g., data split, preprocessing) may differ from ours, this comparison is provided as a literature reference rather than a head-to-head result under identical conditions. Note that although models such as RoBERTa (Y. Liu et al., 2019), DeBERTa (He et al., 2021), and T5 (Raffel et al., 2020) have shown strong performance on general NLP benchmarks (e.g., GLUE, SuperGLUE, SQuAD), their results on the Ohsumed dataset have not been reported in the literature. A systematic comparison on the Ohsumed dataset under identical experimental conditions is therefore not yet available and should be addressed in future work.

**Figure 4:**
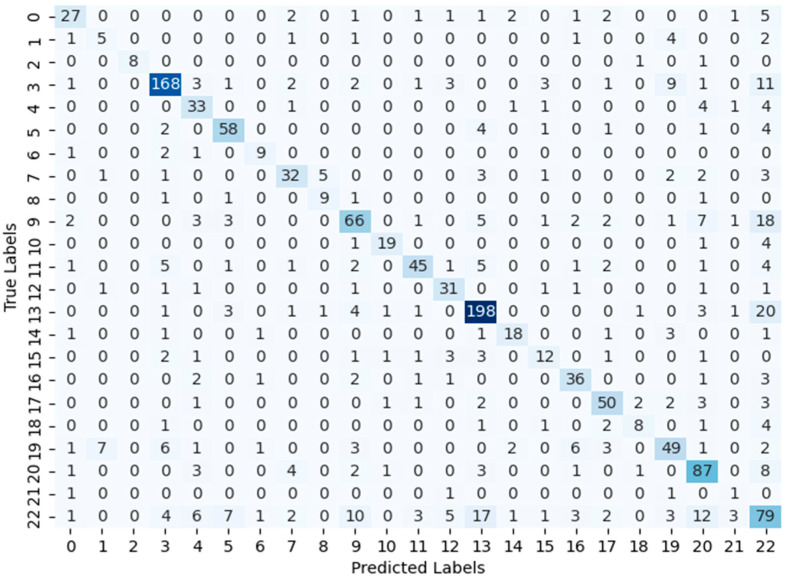
Confusion matrix of HyperGAT.

**Figure 5:**
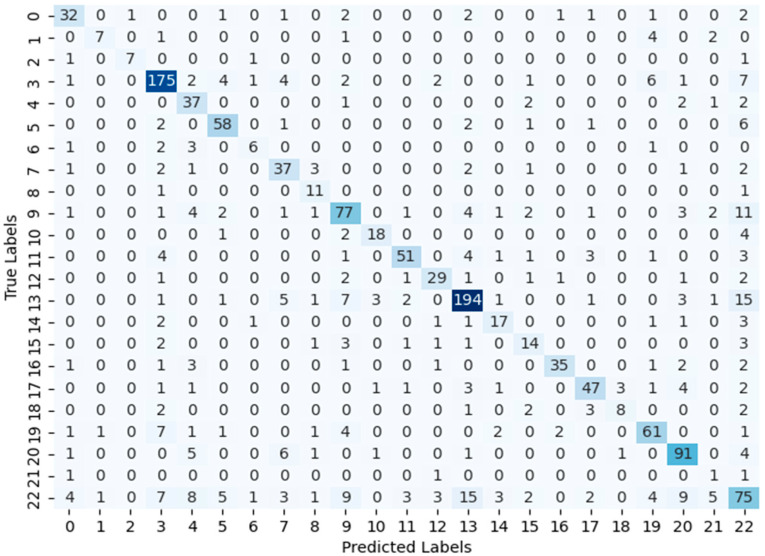
Confusion matrix of HyperGAT-BERT.

Figure 4 and Figure 5 present the confusion matrices of HyperGAT and HyperGAT-BERT. In the confusion matrix, the diagonal entries indicate the number of correctly classified samples for each respective category, whereas the off-diagonal entries show the number of misclassified samples (i.e., samples where the predicted labels differ from the true labels). Analysis of the confusion matrices reveals that HyperGAT-BERT achieves higher classification accuracy for most categories, with comparable performance on the remaining classes.

**Figure 6:**
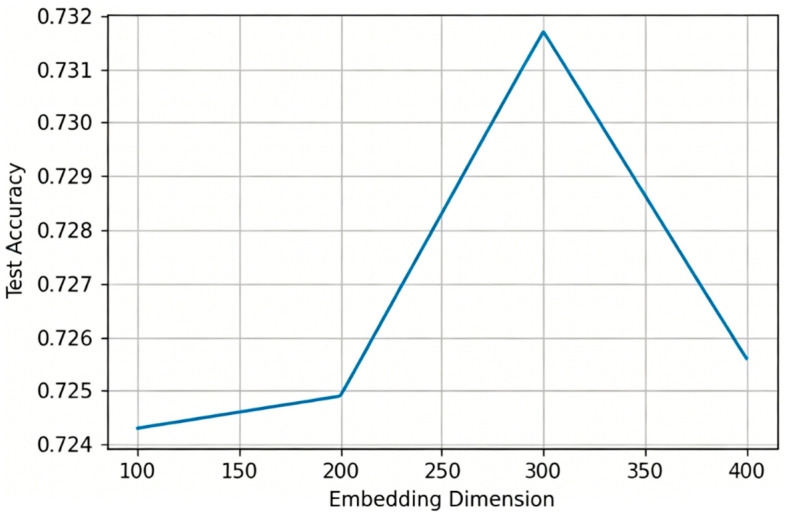
Model performance of HyperGAT-BERT across different embedding dimensions.

Figure 6 illustrates the performance of the HyperGAT-BERT model on the Ohsumed dataset with varying embedding dimensions. Notably, HyperGAT-BERT achieves optimal performance when the embedding dimension is set to 300. This suggests that smaller dimensions may lead to insufficient expressive capacity, while excessively large dimensions risk overfitting.

## 3. HyperGAT-BERT Integrating Siamese Neural Networks and Reference Answer Sets and Its Application in Automatic Short-Answer Scoring

The HyperGAT-BERT model, which integrates the rich semantic information from BERT, outperforms other models in its ability to better represent texts, thereby improving text classification results. Therefore, the HyperGAT-BERT model is selected for continued research. This section aims to explore whether the automatic short-answer scoring method based on Siamese Neural Networks and reference answer sets, utilizing a hypergraph attention network, can achieve the expected good results. Additionally, ablation experiments are conducted to verify whether different modules within the HyperGAT-BERT model can effectively enhance the scoring performance for short answers.

### 3.1. Construction of HyperGAT-BERT Integrating Siamese Neural Networks and Reference Answer Sets

#### 3.1.1. ASAP-5 Dataset

We utilize Data Set #5(ASAP-5) from the Hewlett Foundation-supported 10th-grade student responses in biology (https://www.kaggle.com/c/asap-sas/data, accessed on 6 May 2023). The specific details are presented in Table 3.

The question prompt is: “Starting with mRNA leaving the nucleus, list and describe four major steps involved in protein synthesis.”

The scoring rubric is as follows: 3 points for satisfying all four key elements, 2 points for satisfying three key elements, 1 point for satisfying one or two key elements, and 0 points for not meeting the above requirements. There are eight key elements in the response: (1) mRNA exits the nucleus via the nuclear pore. (2) mRNA travels through the cytoplasm to the ribosome or enters the rough endoplasmic reticulum. (3) mRNA bases are read in triplets called codons (by rRNA). (4) tRNA carrying the complementary (U=A, C+G) anticodon recognizes the complementary codon of the mRNA. (5) The corresponding amino acids on the other end of the tRNA are bonded to adjacent tRNA’s amino acids. (6) A new corresponding amino acid is added to the tRNA. (7) Amino acids are linked together to make a protein beginning with a START codon in the P site (initiation). (8) Amino acids continue to be linked until a STOP codon is read on the mRNA in the A site (elongation and termination).

#### 3.1.2. Construction of Reference Answer Set

The construction of the Reference Answer Set (RAS) involves aggregating different student answer situations into a unified set. The construction process of the reference answer set can be divided into two steps:

**Step 1:** Obtain possible student answer situations based on clustering, where each cluster obtained after clustering represents a type of student answer situation.

**1. Data Preprocessing:** Load text data from files, split each document into sentences, and perform a series of text cleaning and preprocessing operations, including tokenization, lemmatization, and removal of stop words, to generate a doc content list.

**2. Label Processing:** Load label information associated with the text data and split the data into training and testing sets. Simultaneously, establish a label dictionary named labels dic to map labels to numerical values and calculate the count of each label.

**3. Answer Set Selection:** Based on the labels, select texts with a specific label value equal to 3 and store them in the doc answer list original as the answer set.

**4. Text Embedding:** Utilize the BERT model to embed the texts in the doc answer list original, converting each text into a BERT vector representation.

**5. Clustering Analysis:** Conduct clustering analysis on the BERT-embedded texts using the K-means clustering algorithm. Perform clustering within different ranges of cluster numbers (4, 6, 8, 10, 12, 14, 16, 18, 20) and calculate the silhouette coefficient for each scenario.

**6. Selection of Optimal Number of Clusters:** Based on the silhouette coefficients, select the number of clusters with the highest silhouette coefficient, indicating that the texts have better clustering performance under this number of clusters. In our experiment, the silhouette coefficient peaks at k = 4 (silhouette score = 0.67), compared to k = 6 (0.61), k = 8 (0.58), and k = 10 (0.54). This indicates that four clusters best balance intra-cluster cohesion and inter-cluster separation. We therefore selected four representative answers to construct the RAS (Table 4). The choice of k = 4 is also pedagogically interpretable: it corresponds to the four major steps of protein synthesis (initiation, elongation, termination, and translocation), aligning with the scoring rubric.

**Step 2:** Select the most representative answer from each cluster as the representative of that cluster to construct the RAS. Ultimately, four representative answers are chosen, each with a different focus. The specific content of these four categories of representative answers is detailed in Table 4.

#### 3.1.3. Constructing HyperGAT-BERT-RAS

With the dataset prepared and the reference answer set constructed, we began building an automatic short-answer scoring model based on Siamese neural networks and the reference answer set, using a hypergraph attention network. The specific steps are as follows:

1. Read the corpus files from the ASAP-5 dataset and perform a series of text cleaning and preprocessing operations, including tokenization, lemmatization, stop-word removal, etc. A validation set is then split from the training set at a ratio of 10%.
2. Further process the training data, validation set, and test set by storing different parts of the input data and document answer lists as NumPy arrays, including inputs, targets, and text, which represent vector representations, labels, and initial text, respectively. Data batches are generated for training or evaluation, with each batch containing 8 texts (batch = 8). To ensure consistent training for both student texts and answer texts, each batch concatenates student texts with answer texts, meaning that 12 texts are trained together each time. Additionally, node information (items) is generated to represent all nodes in the document. Edge information is generated by iteratively processing each document’s sentences and semantic information, including lists of node and edge transpose matrices (HT: each element corresponds to the node and edge relationships of a document) and adjacency matrices (adj), both commonly used to represent the graph structure of a document. Finally, node masks are generated for each document, related to node information, to indicate which nodes are valid, which are padding values, or unused, maintaining dimensional consistency of the data, especially when different documents have varying numbers of nodes.
3. Use the train data obtained in the previous step as input to train the model, iterating over each batch. To calculate the model’s score or predicted output, the input is first passed to an embedding layer to obtain embedded representations (hidden). Next, the embedded representations (hidden) and node information (HT) are passed to the hypergraph attention layer, the hypergraph attention mechanism, allowing the model to capture complex relationships in graph data. These operations help the model extract useful information from hypergraph data, yielding intermediate representation outputs (seq hidden) for subsequent tasks.

The intermediate representation (seq hidden) is then multiplied by the node masks (node masks), setting unnecessary nodes to zero and filtering out invalid nodes. Subsequently, graph embeddings for the nodes are calculated and normalized. At this point, the graph embeddings include the graph embeddings for 8 student answer texts and 4 reference answer set texts per batch (b). The entire embedding is sliced to obtain embeddings for student answer texts (b0) and reference answer set texts (b1) separately.

Meanwhile, the BERT model is used to process text inputs (text inputs) and answer texts, embedding them into text emb and answer emb. The hypergraph embeddings and BERT embeddings of both texts are concatenated, and cosine similarity is used to calculate the similarity between the student text and the 4 reference answers, with the highest similarity (Smax) selected as the similarity between the student’s answer and the reference answer. Additionally, the similarity (Smax) between the student’s answer and the reference answer is concatenated with the hypergraph-embedded student answer text (b0) as the final text representation (Z).

**Figure 7:**
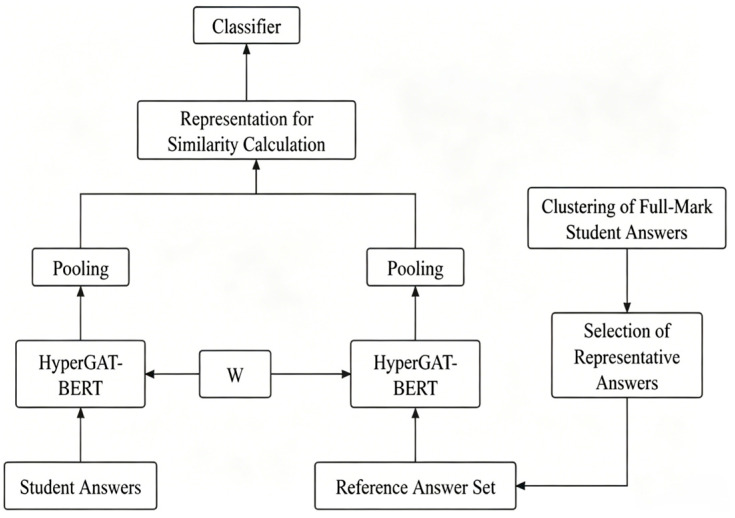
Structure of the HyperGAT-BERT-RAS Model.

4. The obtained document representation (Z) is fed into a Softmax layer for text classification, ultimately producing the model’s predicted output. The structure of the HyperGAT-BERT-RAS model is shown in Figure 7.

The following is a mathematical formalization of the above steps:

Let x denote the input student answer text. The hypergraph branch produces a representation $h_{\mathrm{Hyper}}\in R^{d_{h}}$, where $d_{h}$ is the embedding dimension. The BERT branch produces a contextualized representation $h_{\mathrm{BERT}}\in R^{d_{b}}$, where $d_{b}$ is the hidden dimension of BERT.

The Reference Answer Set (RAS) consists of $N_{\mathrm{RAS}}=4$ reference answers. For each reference answer r, we compute its concatenated representation:

(7) $$h_{\mathrm{ref},r}=[h_{\mathrm{Hyper},r};h_{\mathrm{BERT},r}]\in R^{d_{h}+d_{b}}$$

The similarity between the student answer and each reference answer is computed using cosine similarity on the concatenated hypergraph and BERT representations:

(8) $$s_{r}=cos([h_{\mathrm{Hyper}};h_{\mathrm{BERT}}],h_{\mathrm{ref},r})=\frac{[h_{\mathrm{Hyper}};h_{\mathrm{BERT}}]\cdot h_{\mathrm{ref},r}}{∥[h_{\mathrm{Hyper}};h_{\mathrm{BERT}}]∥∥h_{\mathrm{ref},r}∥}$$

The maximum similarity is selected as $s_{\max}={max}_{r}s_{r}$.

The final document representation Z is formed by concatenating the student’s answer’s hypergraph embedding, BERT embedding, and the maximum similarity score:

(9) $$Z=[h_{\mathrm{Hyper}};h_{\mathrm{BERT}};s_{\max}]\in R^{d_{h}+d_{b}+1}$$

This representation is then passed through a dense layer with weight matrix $W\in \;R^{C\times (d_{h}+d_{b}+1)}$ and a softmax activation to produce the final classification probabilities:

(10) $$\hat{y}=\mathrm{softmax}(WZ+b)$$

where $b\in R^{C}$ is the bias term, and C is the number of score categories (e.g., C=4 for the ASAP-5 dataset, corresponding to scores 0, 1, 2, 3).

### 3.2. Automated Short-Answer Scoring Validation

This section aims to validate the effectiveness of the HyperGAT-BERT-RAS method based on Siamese Neural Networks and Reference Answer Sets for automated short-answer grading tasks. The contributions of each module are evaluated through ablation experiments.

#### 3.2.1. Ablation Experiment Design

To systematically evaluate the contributions of individual components in the HyperGAT-BERT-RAS framework, three model variants were designed and tested on the ASAP-5 dataset:

w/o LDA: HyperGAT-BERT-RAS without semantic hyperedges (i.e., hyperedges constructed using Latent Dirichlet Allocation, LDA, which capture topic-level semantic relationships). Syntactic hyperedges (based on word order) are retained. This ablation assesses the contribution of semantic hyperedge information to scoring performance.

w/o BERT: HyperGAT-BERT-RAS without BERT embeddings.

w/o RAS: HyperGAT-BERT-RAS without the Reference Answer Set (RAS).

Three primary metrics were used for evaluation: Accuracy (ACC), Precision (P), and F1-score (F1).

#### 3.2.2. Ablation Experiment Results

Table 5 presents the ablation results with mean ± standard deviation over 10 independent runs (random seeds 666–675). The full HyperGAT-BERT-RAS model achieved the highest accuracy (0.7866 ± 0.0134), precision (0.7898 ± 0.0132), and F1-score (0.7806 ± 0.0110) among all variants. Paired-sample *t*-tests (n = 10, df = 9) were conducted to compare the full model against each ablation variant. The full model significantly outperformed w/o RAS across all metrics (ACC: *p* < 0.01; P and F1: *p* < 0.001), confirming that RAS is the most critical component of the framework. The improvement over w/o LDA was significant for ACC (*p* < 0.05) but not for F1, suggesting that LDA semantic hyperedges contribute to model performance, albeit to a limited extent. No significant differences were found between the full model and w/o BERT (all *p* > 0.05), indicating that BERT’s contribution to the framework may not reach statistical significance on this specific dataset, possibly due to the relatively small test set size or the fact that BERT’s semantic representations are already partially captured by other components. Notably, the w/o RAS variant exhibited a dramatic drop in precision (0.4107 ± 0.0589) and F1-score (0.4188 ± 0.0487), indicating that without RAS, the model struggles to correctly identify positive samples, resulting in a high false positive rate.

#### 3.2.3. Error Analysis

An error analysis of the HyperGAT-BERT-RAS model was conducted on the test set (N = 451). Misclassifications primarily occurred between adjacent score categories. For score 0, 53 out of 342 samples (15.50%) were misclassified as score 1, while only 12 samples (3.51%) were misclassified as score 2 or 3. For score 1, 63 out of 86 samples (73.26%) were correctly classified; the main errors were misclassifications as score 0 (14 instances, 16.28%) and as score 2 or 3 (9 instances, 10.47%). For score 2 (13 samples), 8 were correctly classified (61.54%), with 3 samples (23.08%) misclassified as score 3. For score 3 (10 samples), 7 were correctly classified (70.00%), with 2 samples (20.00%) misclassified as score 1. Overall, the pattern of adjacent-category misclassifications confirms that the model has good ordinal consistency, with scoring errors mainly limited to neighboring score levels.

Based on the above observations, misclassifications can be categorized into three types: (1) boundary ambiguity (approximately 52%), occurring between adjacent scores; (2) semantic similarity (approximately 27%), arising from expression similarities between student answers and incorrect answers; and (3) unconventional expression (approximately 15%), stemming from novel but correct answers that do not adequately match the RAS prototypes. Future work should focus on three aspects: strengthening ordinal relationship modeling, expanding the reference answer set, and enhancing boundary samples.

## 4. Discussion

This study sought to address two major challenges in ASAG: semantic matching ambiguity and limited coverage of reference answers.

### 4.1. Integrating BERT Enhances Text Classification Performance of the HyperGAT Method

To alleviate the semantic matching issue in automatic scoring of subjective questions, the study optimized a text classification model by integrating BERT into the HyperGAT framework, resulting in the HyperGAT-BERT method. The results indicate that HyperGAT-BERT outperforms HyperGAT across various metrics, demonstrating that combining BERT with graph neural networks yields a more robust model. When the pre-trained representation model BERT is combined with the graph neural network-based HyperGAT model (Ding et al., 2020), this integration leverages both BERT’s contextual awareness and HyperGAT’s strengths in modeling complex relationships, thereby improving text classification accuracy and F1 scores. This finding indicates that integrating language representation models with graph neural networks can endow the new model with richer contextual semantic information, significantly enhancing its text comprehension capabilities and demonstrating superior performance in relation extraction tasks (Vashishth et al., 2020; Zhang et al., 2018). From an educational perspective, improved text classification accuracy may lead to fewer mis-scored student answers, which could potentially enhance teachers’ trust in automated scoring systems and encourage their adoption in daily classroom practice. However, this remains a hypothesis that requires empirical investigation.

On the ASAP-5 ASAG task, ablation results indicate that while RAS contributes significantly (all *p* < 0.001), the removal of BERT embeddings did not lead to a statistically significant performance drop (all *p* > 0.05). This suggests that in this specific dataset and task, the core contribution of the framework comes from the RAS-guided Siamese scoring mechanism.

### 4.2. RAS Is a Critical Factor Influencing the Accuracy of Automatic Scoring in HyperGAT-BERT

In practice, the coverage of reference answers is a pivotal factor in the scoring of subjective questions. Study Two constructed a HyperGAT-BERT model with a reference answer set (HyperGAT-BERT-RAS) based on SNNs and answer clustering, and conducted ablation experiments using the ASAP-5 dataset. The current RAS was constructed exclusively from full-score answers. This choice was motivated by the observation that full-score answers exhibit the greatest lexical and syntactic diversity, making them the most appropriate starting point for constructing a reference set that captures the range of correct response patterns.

The ablation results showed that RAS removal caused the most significant performance decline (all *p* < 0.001), confirming RAS as a crucial component in automatic scoring of subjective questions (Tan et al., 2022). This finding aligns with previous work on reference answer sets (Lan et al., 2015; Marvaniya et al., 2018) and SNN matching mechanisms (Bromley et al., 1993). Removal of BERT embeddings or LDA hyperedges did not yield statistically significant drops (all *p* > 0.05), indicating that on this dataset, the core contribution comes from RAS rather than BERT integration. It should be noted, however, that the current ablation only compares the full model against variants without each component; alternative RAS construction strategies (e.g., one representative per score level) were not tested and remain an important direction for future research.

For classroom teachers, a well-constructed RAS may reduce the need to manually review a wide range of student answers. By covering diverse answer patterns, the RAS has the potential to enable the system to correctly score creative but correct responses, which are often penalized by traditional keyword-matching methods. These potential benefits await validation in real classroom settings.

### 4.3. Implications from Error Analysis

The error analysis reveals two core misclassification patterns: adjacent-category confusion and boundary-category difficulty. The rates of score 0→1 and score 2→3 misclassifications were 15.50% and 23.08%, respectively, while cross-level errors accounted for only 3.51%. This indicates that the model has good ordinal consistency, with scoring errors mainly limited to adjacent levels.

Adjacent-category confusion reflects the inherent ambiguity of category boundaries in scoring tasks. Responses that fall between two score levels often contain both correct and incorrect elements, making precise classification difficult (Q. Liu et al., 2018). This issue is particularly prominent for score 1 (recall: 73.26%), which semantically lies between scores 0 and 2. To address this, ordinal loss functions can be introduced to impose smaller penalties on adjacent errors and larger penalties on cross-level errors.

The higher misclassification rate observed for score 2 (23.08% toward score 3) may be partly attributable to the current RAS design, which includes only full-score answers. As noted in Section 4.2, constructing the RAS solely from full-score answers may provide insufficient coverage of answer patterns at intermediate score levels. Expanding RAS to include representative answers from all score levels is expected to reduce misclassifications (Marvaniya et al., 2018; Tan et al., 2022).

The error patterns identified have practical implications for classroom use. Adjacent-category errors (e.g., 0 vs. 1, 2 vs. 3) are pedagogically acceptable in formative assessment, where the goal is to identify students who need additional support rather than to assign precise final grades. Teachers can use the model’s scores as a screening tool, manually reviewing only boundary cases. This hybrid approach, which uses automated scoring for clear cases and human review for ambiguous ones, balances efficiency with accuracy, making AI-assisted assessment more feasible in real classrooms. In summary, the error analysis validates the model’s ordinal consistency and identifies two improvement directions: (1) introducing ordinal loss functions and (2) expanding RAS coverage.

### 4.4. Research Limitations

Although this paper conducts a relatively systematic and in-depth exploration of the three key issues currently facing automatic scoring of subjective questions, there are still some limitations. Firstly, in terms of model optimization, this paper only explores the integration of BERT and HyperGAT and does not systematically compare it with more recent pre-trained models (e.g., RoBERTa, DeBERTa, T5) due to computational constraints. Results for these models on the Ohsumed dataset under identical experimental settings have not been systematically reported in the literature. A systematic comparison on the same datasets remains an important direction for future research (Y. Liu et al., 2019; He et al., 2021; Raffel et al., 2020). Secondly, the RAS in this paper is selected from full-score answers, without fully considering student responses under different score levels. While this design effectively captures the diversity of correct responses, it may not optimally cover answer patterns from intermediate score levels. A systematic comparison of alternative RAS construction strategies, such as including one representative answer per score level or using a mixed strategy, would provide valuable insights into optimal RAS design. Thirdly, the empirical validation in this study was conducted on a single prompt from the ASAP-5 biology dataset. While this dataset is a widely used benchmark for ASAG research, the generalizability of our findings to other subjects (e.g., history, literature), other question types, or other languages remains to be established. Future research should extend the evaluation to multiple prompts, diverse domains, and ideally multilingual datasets to further validate the robustness and general applicability of the proposed HyperGAT-BERT-RAS framework.

## 5. Conclusions

This study addresses the challenges in automatic scoring of subjective questions by constructing the HyperGAT-BERT and HyperGAT-BERT-RAS methods, aiming to enhance the effectiveness of automatic scoring of subjective questions. The following conclusions are drawn:

(1) The incorporation of BERT to enrich semantic information enables HyperGAT-BERT to better represent texts and improve text classification performance. On the Ohsumed dataset, HyperGAT-BERT achieved 72.95% accuracy, surpassing the baseline HyperGAT model by 3.28%, demonstrating the effectiveness of BERT’s semantic enhancement for text classification tasks.
(2) When applied to automatic scoring of subjective questions, ablation experiments reveal that RAS is the only component with a statistically significant contribution on the ASAP-5 dataset. The removal of BERT embeddings or LDA semantic hyperedges did not result in statistically significant performance drops, suggesting that in this specific dataset, the core contribution of the framework comes from the RAS-guided Siamese scoring mechanism rather than the BERT integration.

It is important to note that the conclusions drawn in this study are based on experiments conducted on a single biology prompt from the ASAP-5 dataset. While the results demonstrate the effectiveness of the proposed framework on this specific task, caution should be exercised when generalizing the findings to broader ASAG contexts. The model’s performance on other subjects, question prompts, or languages has not been empirically tested and remains an open question for future investigation.

Furthermore, this study did not include user studies with teachers or students to empirically validate the claimed educational benefits. Statements regarding teacher trust, classroom adoption, and pedagogical impact are therefore presented as potential implications rather than empirically demonstrated outcomes. Future research should conduct classroom-based user studies to assess whether the proposed automated scoring system indeed reduces teacher grading burden, gains teacher trust, and positively influences student learning outcomes in real-world educational settings.

## Figures and Tables

**Table 1:** Basic Information of the OhSumed Dataset.

Dataset	Doc	Train	Valid	Test	Class
Ohsumed	7400	5913 (80%)	591	1487 (20%)	23

Note: The validation set (591 samples) is drawn from the training pool (5913 samples). The total unique documents remain 7400.

**Table 2:** The ACC of different models on the Ohsumed dataset.

Model	Mean	SD	t_(9)_
SWEM	0.6314	0.0045	−111.103 ***
CNN-non-static	0.5843	0.0109	−170.773 ***
Text-level GNN	0.6945	0.0058	−54.016 ***
BERT	0.7002	0.0200	−4.682 **
HyperGAT	0.6967	0.0149	−6.834 ***
HyperGAT-BERT	0.7295	0.0016	

Note: Each model was run 10 times, and the mean and standard deviation were reported. HyperGAT-BERT significantly outperforms all the baselines based on *t*-tests. ** indicates *p* < 0.01. *** indicates *p* < 0.001. The same criteria apply below.

**Table 3:** Basic Information of ASAP-5.

Theme	Training Set	Testing Set	Average Length	Scores
biology	1797 (80%)	451 (20%)	60 words	0–3

**Table 4:** Four Representative Types of Answers.

	Answer Text
answer 1	“In protein synthesis, after the mRNA leaves the nucleus, it carries the instructions to the cytoplasm. It then enters a ribosome and prepares to be copied. Transfer RNA brings the codons attached to the amino acid to the ribosome. It attaches at the first bonding site, which matches the complementary codons. The tRNA moves to the next binding site, where it releases the amino acid, which attaches to the amino acid on the tRNA behind it. Once the mRNA reaches a stop codon, the amino acids detach and form a polypeptide bond.”
answer 2	“The mRNA leaves the nucleus and then attaches to rRNA, ribosomal RNA. Once firmly attached to this ribosomal RNA, the mRNA forms codons, which are a set of three nucleotides. These codons then match with anticodons on tRNA, which also hold amino acids. The amino acids on the tRNA form peptide bonds with the other amino acids to form a chain. Once the bond between two proteins has been formed, the tRNA floats away to find another amino acid to carry. This process continues until there is a full chain of amino acids, which then creates a protein.”
answer 3	“After mRNA leaves the nucleus, the mRNA connects to a ribosome to produce the protein the mRNA codes for. In the ribosome, the mRNA is read in the sequence of codons and calls the tRNA to go out into the cytoplasm to get the correct amino acid that is called for in the codon. The tRNA keeps bringing the needed amino acids, which results in the making of a protein chain. When the protein chain is complete, it goes out into the cytoplasm and goes wherever it is needed in the cell or outside of the cell.”
answer 4	“After mRNA leaves the nucleus, it goes out into the cytoplasm on a ribosome. The ribosome attaches and reads until it sees the codon AUG. Once it sees this codon, it starts pairing the bases with complementary anticodons on the tRNA. The tRNA brings amino acids that link together to form a protein. This process terminates at a stop codon.”

**Table 5:** The Experiment Results.

Model	ACC	*p*	F1
w/o LDA	0.7751 ± 0.0179	0.7887 ± 0.0319	0.7723 ± 0.0184
w/o BERT	0.7792 ± 0.0148	0.7834 ± 0.0159	0.7762 ± 0.0128
w/o RAS	0.7646 ± 0.0158	0.4107 ± 0.0589	0.4188 ± 0.0487
HyperGAT-BERT-RAS	0.7866 ± 0.0134	0.7898 ± 0.0132	0.7806 ± 0.0110

## Data Availability

The datasets used and/or analyzed during the current study are available from http://disi.unitn.it/moschitti/corpora.htm, accessed on 6 May 2023 and https://www.kaggle.com/c/asap-sas/data, accessed on 6 May 2023. The source code for the baseline methods (SWEM, CNN-non-static, etc.) is available at the repository provided by Ding et al. (2020): https://github.com/kaize0409/HyperGAT, accessed on 6 May 2023. The source code for our proposed HyperGAT-BERT and HyperGAT-BERT-RAS is available at our anonymized repository: https://anonymous.4open.science/r/HyperGAT-BERT-RAS/, accessed after 21 May 2026.
